# The Influence of Recovery and Training Phases on Body Composition, Peripheral Vascular Function and Immune System of Professional Soccer Players

**DOI:** 10.1371/journal.pone.0004910

**Published:** 2009-03-18

**Authors:** Simon Reinke, Tim Karhausen, Wolfram Doehner, William Taylor, Kuno Hottenrott, Georg N. Duda, Petra Reinke, Hans-Dieter Volk, Stefan D. Anker

**Affiliations:** 1 Division of Applied Cachexia Research, Department of Cardiology, Charité - Universitätsmedizin Berlin, CVK, Berlin, Germany; 2 Berlin-Brandenburg Center for Regenerative Therapies (BCRT), Charité - Universitätsmedizin Berlin, Berlin, Germany; 3 Julius Wolff Institut and Center for Musculoskeletal Surgery, Charité -Universitätsmedizin Berlin, Berlin, Germany; 4 Department of Sportwissenschaft, Martin – Luther - University Halle-Wittenberg, Halle-Wittenberg, Germany; 5 Department of Nephrology and Internal Intensive Care, Charité Universitätsmedizin Berlin, CVK, Berlin, Germany; 6 Institut of Medical Immunology, Charité Universitätsmedizin Berlin, CCM, Berlin, Germany; Karolinska Institutet, Sweden

## Abstract

Professional soccer players have a lengthy playing season, throughout which high levels of physical stress are maintained. The following recuperation period, before starting the next pre-season training phase, is generally considered short but sufficient to allow a decrease in these stress levels and therefore a reduction in the propensity for injury or musculoskeletal tissue damage. We hypothesised that these physical extremes influence the body composition, blood flow, and endothelial/immune function, but that the recuperation may be insufficient to allow a reduction of tissue stress damage. Ten professional football players were examined at the end of the playing season, at the end of the season intermission, and after the next pre-season endurance training. Peripheral blood flow and body composition were assessed using venous occlusion plethysmography and DEXA scanning respectively. In addition, selected inflammatory and immune parameters were analysed from blood samples. Following the recuperation period a significant decrease of lean body mass from 74.4±4.2 kg to 72.2±3.9 kg was observed, but an increase of fat mass from 10.3±5.6 kg to 11.1±5.4 kg, almost completely reversed the changes seen in the pre-season training phase. Remarkably, both resting and post-ischemic blood flow (7.3±3.4 and 26.0±6.3 ml/100 ml/min) respectively, were strongly reduced during the playing and training stress phases, but both parameters increased to normal levels (9.0±2.7 and 33.9±7.6 ml/100 ml/min) during the season intermission. Recovery was also characterized by rising levels of serum creatinine, granulocytes count, total IL-8, serum nitrate, ferritin, and bilirubin. These data suggest a compensated hypo-perfusion of muscle during the playing season, followed by an intramuscular ischemia/reperfusion syndrome during the recovery phase that is associated with muscle protein turnover and inflammatory endothelial reaction, as demonstrated by iNOS and HO-1 activation, as well as IL-8 release. The data provided from this study suggest that the immune system is not able to function fully during periods of high physical stress. The implications of this study are that recuperation should be carefully monitored in athletes who undergo intensive training over extended periods, but that these parameters may also prove useful for determining an individual's risk of tissue stress and possibly their susceptibility to progressive tissue damage or injury.

## Introduction

In modern sports medicine, little is known about the relationship between functional capacity, such as oxygen consumption or peripheral blood flow, physical stress, and the parameters of immune activation. Many studies have focussed on aerobic-anaerobic endurance [1], [2], the aerobic-anaerobic threshold [1], [2], [3] or muscular regeneration [4], [5], [6], resulting in an important understanding for the enhancement of physical performance after short or intermediate-term training [7]. Professional athletes, however, are generally exposed to extended periods of high physical stress with only short phases of recovery and recuperation. Such athletes seem to be prone to injuries of the musculoskeletal system due to the imbalance of physical strain to recuperation [8]. Professional soccer players have a long competitive season, followed by a short break of only three to four weeks, before commencing endurance training prior to the start of the following season. This recuperation period is considered short, and it is therefore unknown whether the reduction of physical stress levels are sufficient to reduce the propensity for injury or musculoskeletal tissue damage. Indeed, many muscular and ligamentous injuries occur without the influence of opponents or even a ball [9]. The high levels of injury in elite soccer players, might indicate that thorough recovery is not actually achieved between playing seasons.

Physical stresses have additionally been shown to be activators for many processes of the immune system [10], [11], including the up-regulating of e.g. leukocytes, neutrophiles, granulocytes and monocytes, after acute physical stress such as high level training or competitive performances [12], [13]. In this context, the endothelium plays a central role in the regulation of peripheral blood flow and vascular resistance by generating nitric oxide (NO) [14]. Furthermore endothelial cells are activated during acute inflammation to support repair mechanisms of injured tissue [15]. The ability to detect an individual's exposure to physical and/or psychological stress would therefore appear to be an important prerequisite for monitoring their propensity for injury.

In their role as mediators at critical points during acute and chronic inflammation, endothelial cells can change their properties, amongst others, to enable:

i) the release of cytokines such as IL-8 to support the chemotaxis of leukocytes to the inflamed tissue [16],
ii) the up-regulation of NO (via iNOS) regulating blood flow [17], [18],
iii) the up-regulation of adhesion molecules (ICAM, VACAM etc.) to support leukocyte sticking and transmigration from vessels to tissue [19],
iv) the up-regulation of the stress protein, hemoxygenase-1 (HO-1) that metabolises the pro-inflammatory and toxic hem (e.g. as product of muscle protein degradation) into regulatory down-stream products biliverdin (converted into bilirubin), free iron (inducing ferritin), and carbon monoxide (enhancing blood flow and protecting endothelial cells) [20].

The quantification of such processes or markers would therefore seem to allow indirect access to the level of physical stress. Until now, most studies in this area have examined the acute reaction of the immune system and endothelium after sports activity. However, the effect of periods of high physical stress followed by only short phases of recovery has not been well documented. Therefore we hypothesised that the physical extremes imposed upon these athletes over prolonged periods of time influence the body composition, blood flow, and endothelial/immune function, but that the recuperation is maybe insufficient to allow a reduction of tissue stress damage. The aim of this study was to examine how extreme phases of physical strain influence body composition, peripheral vascular blood flow and endothelial/immune function by examining professional soccer players at the end of a season, after the season intermission, and following the start-up training for the new season.

## Methods

### Subjects and study protocol

10 first team soccer players (aged 20 to 36 years, 9 Caucasian, 1 non-Caucasian) from the German Bundesliga were studied. All participants gave written informed consent and the study was approved by the local Ethics Committee of the Charité – Universitätsmedizin Berlin. Clinical evaluation included heart rate, blood pressure, ECG, electrolytes, glucose concentration, kidney function and liver function (Table 1).

**Table 1:** Baseline characterisation of subjects.

Parameters	mean±SD
age	25.3±5.1
height (cm)	184.2±5.9
weight (kg)	90.1±5.6
body mass index	26.1±1.0
total lean mass (kg)	74.4±4.2
total fat mass (kg)	10.3±5.6
body fat (%)	11.9±6.2
VO_2_-max (ml/min/kg)	51.2±4.6
haemoglobin (g/dl)	14.5±1.0
haematocrite (%)	42.5±2.8
potassium (mmol/l)	3.9±0.2
sodium (mmol/l)	141.1±1.8
serum creatinine concentration (µmol/l)	100.1±10.8
leucocytes count (/nl)	4.9±1.5

The first assessment, representing the phase of maximal exhaustion, was performed at the end of the regular season in May 2005. In this regular playing season of 2004/2005, the average number of first team games per participant was 23.6 from a maximum of 34 league games. After this playing season, and at the end of the recovery holiday period of four weeks, a second series of tests was performed in June 2005. During this period of recuperation, the players were advised to avoid physical activity for at least three weeks. In the last week of their holidays they had to perform aerobic running training for not more than one hour per day.

The subjects were studied a final time after a pre-season training of five weeks, which represented the preparation period for the following season (August 2005). During this period, the players attended a high intensity endurance and power training, as well as training of different forms of football specific skills. The amount of training ranged from two to three sessions per day.

At each measurement time point, we performed a dual energy X-ray absorptiometry (DEXA) scan to examine body composition, as well as venous occlusion plethysmography to determine the peripheral blood flow and vascular capacity. Furthermore, we took venous blood samples for the series of serum parameter tests.

### Blood samples

On each test day, blood samples were taken in the morning between 0800h and 1000h in a quiet room after a period of rest for at least 15 minutes. Full blood count, clinical chemistry (including ferritin, bilirubin & nitrates) and different parameters of immune and endothelial function (e.g. interleukins, TNF, nitrate, leucocyte cell count) were assessed. Full blood count and clinical chemistry were measured in plasma and serum samples according to the laboratory standard operating procedures.

Cytokines (TNF, IL-6, total IL-8, CRP) were measured in plasma samples by using the semiautomatic system Immulite (Siemens/DPC, Bad Nauheim, D), according to the recommendations of the manufacturer.

### Body composition

Dual energy X-ray absorptiometry (DEXA) was performed using a lunar prodigy model (General Electric, Madison, Wisconsin, USA) at two different x-ray energies of 38 keV and 70 keV. Total mass, lean mass, body fat, and bone mineral content were assessed for the whole body, as well as for specific regions of interest (e.g. upper and lower extremities). Each scan took about seven minutes and scan data was analysed using Lunar “en Core 2002” software. The mean radiation dose per scan for body composition is reported to be 0.4 µSv with a measurement precision of 99% (Lunar Radiation Company).

### Peripheral blood flow and vascular capacity

Peripheral blood flow and vascular capacity was investigated using venous occlusion plethysmography performed with an EC 6 plethysmograph (Hokanson Inc., Bellevue, USA) system. The subjects rested in a supine position for at least 15 minutes, and forearm blood flow was then determined using a mercury-in-silastic strain gauge (Hokanson Inc., Bellevue, USA). A cuff around the right upper arm was connected to a rapid inflation pump with an air source and solenoid valves, used to inflate and deflate the occlusion cuff rapidly to the required pressure of 40 mmHg. To measure the peak forearm blood flow, the cuff was inflated to suprasystolic pressure (30 mm HG above systolic blood pressure) for 3 minutes. The blood flow was measured after release of the cuff in ten second intervals for at least two minutes. The highest flow results were considered to represent peak forearm blood flow.

Flow dependent flow (FDF), a non-invasive method to estimate endothelium dependent vasodilatation, was assessed at the forearm as an estimate of endothelium dependent vasodilator capacity [21]. A second sphygomanometer cuff was placed distal to the strain-gauge and inflated to suprasystolic levels for 2 minutes. After sudden deflation of this cuff, the increase of shear stress due to increased flow in the brachial artery causes endothelium dependent NO release. Blood flow was then measured every 10 seconds for a period of 90 seconds. All results for plethysmography are given in millilitres per 100 ml tissue per minute (ml/100 ml/min).

All the above tests were performed in a dedicated quiet room between 0900h and 1200h to prevent data bias from noise and circadian rhythms.

For characterisation of the subjects' metabolic performance, a single cardiopulmonary exercise test was performed using an Innocor™ system (Innovision, Odense, Denmark) and a treadmill HP cosmos® (HP Cosmos sports & medical GmbH, Nussdorf-Traunstein, Germany). The exercise protocol used for cardiopulmonary exercise testing involved running on a treadmill whilst oxygen consumption and anaerobic threshold were investigated (Table 2). The highest value measured for oxygen consumption was taken as the maximum cardiopulmonary capacity and anaerobic threshold was assessed using the v-slope method [22], [23].

**Table 2:** Protocol of exercise test.

Stage	Gradient (°)	Speed (m/s)	Time (min)
0	3	0.0	0–2
1	3	3.0	2–4
2	3	3.4	4–6
3	3	3.8	6–8
4	3	4.2	8–10
5	3	4.6	10–12
6	3	5.0	12–14
7	3	5.4	14–max. exhaustion

### Statistical analysis

All results are presented as a mean value±SD. The Wilcoxon signed rank test was applied to scan the variables between each measurement and the spearman rank correlation was used for non-parametric correlation analyses between different parameters. The statistical analyses were performed using StatView 4.5 (Abacus Concepts Inc., Berkeley, USA). Statistical significance was indicated at P<0.05.

## Results

We studied the effect of training and recovery on parameters of body composition, endothelial function and markers of the immune system in 10 professional first team soccer players (aged 20 to 36 years) of the German Bundesliga. All clinical parameters: heart rate, blood pressure, serum electrolytes, glucose concentration, kidney function and others showed normal values (Table 1). The values for a single player were not available in the final measurement session due to a stomach virus.

### Changes in body composition

At the end of the regular playing season, players showed a mean body weight of 90.1±5.6 kg, with a body fat content of 11.9±6.2% and lean body mass of 74.4±4.2 kg.

**Figure 1:**
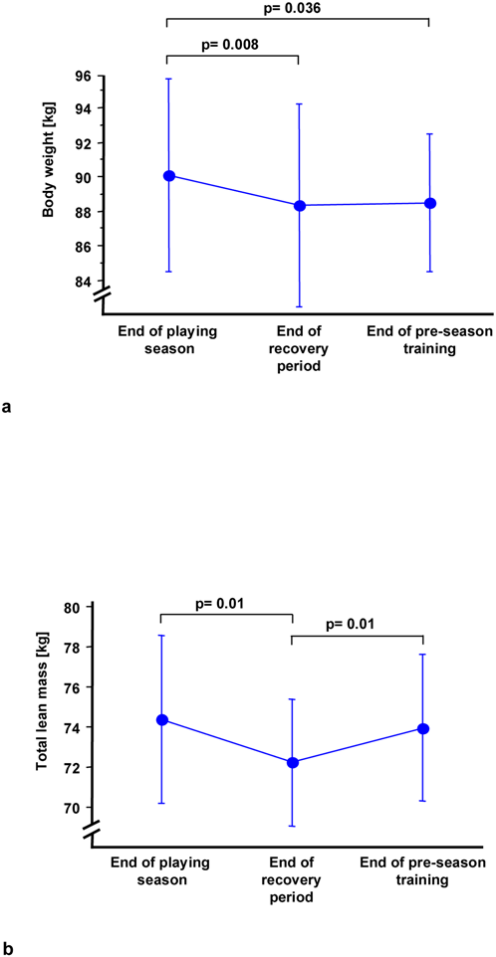
Changes in body weight (a) and lean mass (b) during different phases of physical strain.

Significant changes in body composition were observed during the test stages (Figure 1). A significant reduction in body mass was found at the end of the recovery phase, followed by a slight increase after pre-season training (end of regular season: 90.1±5.6 kg vs. after 4 weeks recuperation: 88.3±5.9 kg vs. after pre-season training: 88.5±4.0 kg). This could be accounted for in the reduction of lean body mass (74.4±4.2 kg vs. 72.2±3.2 kg vs. 73.9±3.7 kg). Fat mass increased during the recovery phase and decreased during the pre-season training (10.3±5.6 kg vs. 11.1±5.4 kg vs. 9.4±4.6 kg).

**Figure 2:**
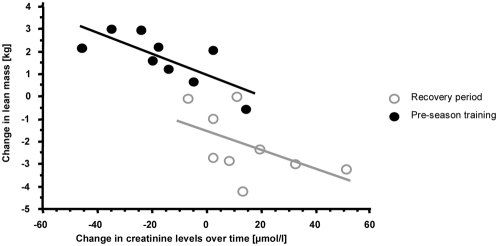
A significant inverse correlation was found between the total lean mass and increased/decreased levels of creatinine over the time period from the end of playing season to the end of recovery, as well as from recovery to the end of pre-season training.

In parallel to changes in the body composition, enhanced serum creatinine levels were observed at the end of the recovery phase. Blood urea remained unchanged at this time. Moreover, a change of lean mass showed a close inverse relationship to serum creatinine concentrations in the different phases (playing season to recovery: r = −0.59; p<0.09 and recovery to pre-season: r = −0.75; p = 0.04) (Figure 2). At the end of the pre-season training, both parameters reached baseline (assumed to be end of playing season) levels again. Although no statistical significance was observed between the time points, the levels of creatine kinase (293.7±163.7 U/L at the end of the season, 348.8±182.9 U/L after recuperation, and 332.1±148.3 U/L during pre-season training) demonstrated that the largest muscle turnover did coincide with the period of recuperation when the players lost most lean body mass.

### Changes in blood flow

**Figure 3:**
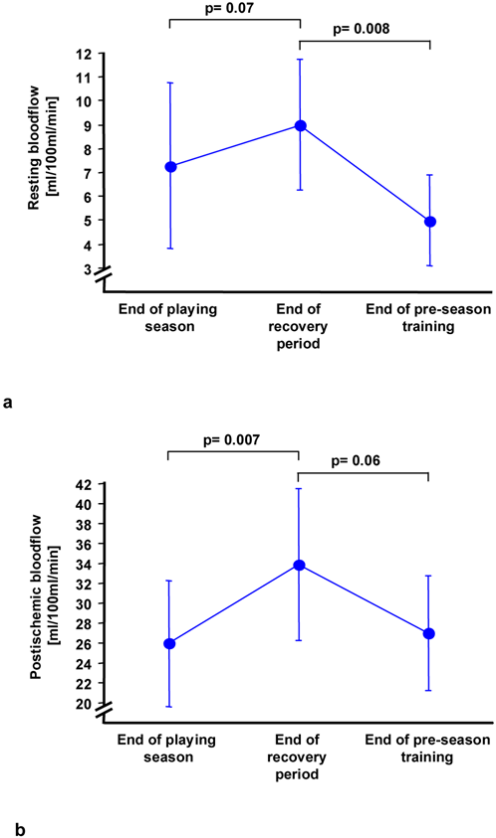
Enhanced resting and postischemic blood flow during the recovery period.

Examination of the peripheral venous blood flow showed significant differences between the phases of physical stress and recovery. Both the resting and post-ischemic blood flows were subnormal in the athletes at the end of the playing season, reaching levels comparable with mild chronic cardiac failure patients [21], [24] suggesting compensated hypo-perfusion. Remarkably, we found an increase of resting blood flow by 23% to the measured baseline level (Baseline: 7.3±3.4 vs. end of recovery 9.0±2.7 ml/100 ml/min) after the recuperation phase. After pre-season training for the following season, resting blood flow showed even lower levels when compared with baseline values (baseline 7.3±3.4 vs. end of pre-season training: 5.0±1.9 ml/100 ml/min). Furthermore, post-ischemic blood flow was elevated by 32% after the recovery period compared with season end (baseline: 25.9±6.3 vs. end of recovery 34.0±7.6 ml/100 ml/min), but reached baseline levels once again after pre-season training (Figure 3).

### Changes in endothelial and immune function

**Figure 4:**
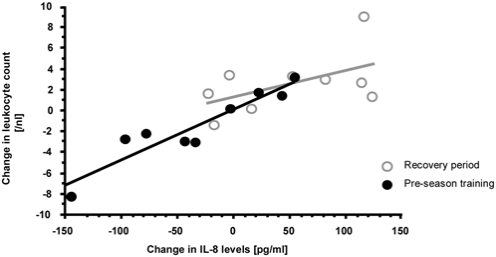
A significant correlation between IL-8 levels and leukocyte count demonstrated a recovery from stress-related leukopenia from the playing season to the recuperation phase, as well as a return to lower leukocyte counts after the stressfull phase of pre-season training.

**Figure 5:**
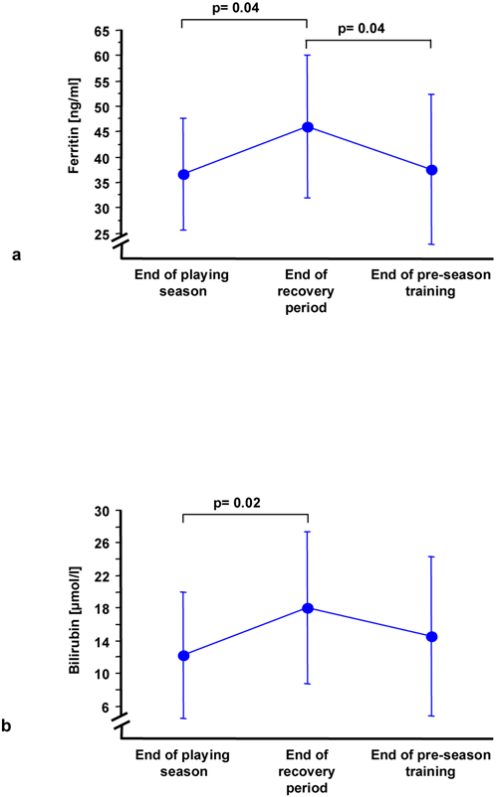
Increased (a) ferritin and (b) bilirubin concentrations during recovery both demonstrate enhanced HO-1 activity.

A significant increase of nitrate was observed at the end of the recovery phase (2.3 mg/l±1.2 vs. 4.2 mg/l±1.0; p<0.005) when compared with levels at the end of the previous season. In parallel to enhanced levels of NO, total IL-8 and leukocytes were also upregulated after the recovery phase. This leukocytosis was due to an increase of granulocyte and monocyte counts, while lymphocytes were decreased during the recovery phase (Table 3). No increases in serum TNF, IL-6 or CRP were observed. Thereafter, a significant correlation between the change in IL-8 levels and changes in leukocyte count was observed from recuperation to pre-season training (Figure 4). Furthermore, a significant up-regulation of both bilirubin and ferritin (Figures 5a & 5b) from normal to enhanced serum levels was observed after the recovery phase, suggesting HO-1 activity. These levels returned to post-season levels after pre-season training.

**Table 3:** Immunological and biochemical paramaters.

Parameters	End of playing season	p-values	End of recovery	p-values	End of pre-season training
Leukocytes (/nl)	4.9±1.5	p = 0.009	7.6±4.0	p = 0.314	6.2±1.5
Lymphocytes (/nl)	1.6±0.4	p = 0.037	1.3±0.4	p = 0.008	1.8±0.4
Monocytes (/nl)	0.37±0.1	p = 0.017	0.55±0.3	p = 0.313	0.44±0.2
Granulocytes (/nl)	2.9±1.1	p = 0.009	5.7±3.5	p = 0.173	3.9±1.3
IL-8 (pg/ml)	106.2±30.1	p = 0.047	156.8±71.5	p = 0.214	125.4±54.2
Creatinine (µmol/l)	100.1±10.8	p = 0.014	114.7±14.8	p = 0.033	98.4±8.1
Bilirubin (µmol/l)	12.2±7.7	p = 0.021	18.1±9.4	p = 0.173	14.5±9.7
Ferritin (ng/ml)	36.6±10.9	p = 0.038	46.0±14.1	p = 0.044	37.5±14.8
Nitrate (mg/l)	2.3±1.2	p = 0.005	4.2±1.0	p = 0.085	2.8±1.4

## Discussion

There is a lack of data on the influence of chronic stress on the physiology of elite athletes. For the first time in this study, we have examined how a cycle of season-long stress, followed by a short recuperation phase and then intensive pre-season training, influences body composition, blood flow and endothelial/immune function in professional soccer players. Following a 4 week recuperation period, a significant decrease of lean body mass but a not unexpected increase in fat mass has been observed. During this recuperation period, raised serum creatinine levels suggest an increase in muscle turnover. These changes were almost completely reversed after pre-season training for the new season. Remarkably, both resting and post-ischemic blood flow were greatly reduced after the playing season's stress, but both parameters increased to normal levels during rest and recuperation. Recovery was also characterized by rising levels of granulocytes, total IL-8, serum nitrate, ferritin, and bilirubin. These data suggest a compensated hypo-perfusion (and hypoxia) during the playing season, followed by an intramuscular ischemia/reperfusion syndrome during the recovery phase that is associated with muscle protein turnover [25] and inflammatory endothelial reaction, as reflected by iNOS and HO-1 activation, as well as IL-8 release.

Periods of intense training were clearly associated with chronic stress and anabolic metabolism in the elite athletes investigated in this study. This was demonstrated by a high lean/total body mass ratio that changed over the observation period, possibly in response to physical or psychological stress. Interestingly, we found vasoconstriction with a reduced peripheral venous blood flow (ave. 25.9 ml/100 ml/min) at the end of the season, levels that are associated with mild cardiac failure patients (11–26 mL/100 mL/min, [21], [24]). In comparison, normal controls have shown blood flow values of 34.2±2.2 mL/100 mL/min [26]. The subjects' response to physical and psychological stress was further established by neutropenia and monocytopenia during the season, resulting in partial insufficiency of innate immunity. The following rest and recuperation period was then associated with a reduction in the levels of stress, as demonstrated by catabolic metabolism connected with vasodilatation as well as an improved venous blood flow. The observed results were somewhat surprising, especially as a higher body weight after recovery and a better blood flow during training phase had been expected.

Nitric oxide (NO) is an endothelium-derived vasodilator that inhibits muscular contraction and therefore produces relaxation in the body. It seems likely that the aforementioned reduction in physical and psychological stress during recovery improved the peripheral blood flow by endothelial release of NO, which was confirmed by the observed increase in nitrates. This is probably the result of enhanced iNOS activity, the most powerful NO releasing enzyme, which is up-regulated following endothelial activation, such as by ischemia/reperfusion injury or inflammation [27], [28], [29]. The parallel up-regulation of IL-8, which supports the recruitment and survival of neutrophil (cells of the innate immune system), was presumably a result of the activation of immune or endothelial cells (since serum TNF, IL-6 and CRP were not increased). This suggests that the athletes possessed a biochemical signature seen typically in ischemia or reperfusion injury [30], [31].

Professional soccer players likely possess a high ratio of fast twitch muscle fibres, which exhibit rapid metabolic adaptation to the mode of training [32], [33]. We therefore speculate that these conditions, together with their intense fitness training, may cause the players to be particularly susceptible to changes in physical strain, and that the loss of muscle mass during recovery might be the physiological response. However, the levels of change of both muscle mass and blood flow were surprising high. It seems reasonable therefore that the reaction to stress results in vasoconstriction and therefore in the adaptation of these fibres to compensated hypoperfusion. Stress relief during recovery seemed to initiate better perfusion, inducing an ischemia/reperfusion reaction and which resulted in a higher muscle turnover, haem release and finally in endothelial activation that was amplified by immune reconstitution. The up-regulation of HO-1 and its cytoprotective down-stream products might be explained as a compensatory mechanism.

Previous studies have demonstrated that the physical performance associated with a single soccer game induces muscle damage and transient inflammation responses for as long as 72 hours post-exercise [13]. Although in this study all serum parameters were taken at least 48 hours after any strenuous exercise, it could be that inflammation was still present. However, hsCRP and IL-6 showed no signs of inflammation at any time point, but also the differences seen in the total levels of leukocytes, particularly granulocytes and monocytes, as well as IL-8, were all at their highest levels during the period of recuperation (Table 3), and not, as expected, during either the playing season or pre-season training. The higher levels of muscle turnover during the recuperation period, therefore only enhance the idea that the immune system is not able to function fully during periods of high physical stress, possibly by down-regulating immune and endothelial function. This could result in the reduced capacity to metabolize any damaged muscle cells [8], [34]. Since no direct proof of muscle damage was available in any player throughout this study, including medical records and the levels of creatine kinase, the observed increase of creatinine did indicate an increased rate of muscle turnover, which was consistent with the reduction of muscle mass during recovery. Here, the immune and endothelial systems might have been more active, allowing any damaged muscle cells to be better metabolized. These results imply that periods of rest can reduce tissue level stress, but suggest that the short recuperation period allowed by top level soccer players might be insufficient to allow complete regeneration. Whether this leads to a more rapid burn out of soccer players, remains to be investigated.

In conclusion, we believe there is vasoconstriction connected with muscular hypoperfusion during intensive periods of training, with reduced immunological repair function due to lower endothelial activity. Whilst levels of stress, and demonstrated by limited immune function and vascular capacity (e.g. peripheral blood flow), appear to be greatly reduced at the end of playing season, stress levels seem to rise again rather rapidly after training recommences, and it therefore appears that longer periods of rest may be required before full recovery is achieved. Although further studies will be needed to validate these results in other sports, particularly in those with similar periods of high physiological and psychological pressure, the results from this study indicate that the inter-individual differences between these parameters might be suitable for assessing an individual's risk of overloading or over stressing at a tissue level.

## References

[1] Lind E, Joens-Matre RR, Ekkekakis P (2005). What intensity of physical activity do previously sedentary middle-aged women select? Evidence of a coherent pattern from physiological, perceptual, and affective markers.. Prev Med.

[2] Hollmann W (2001). 42 years ago–development of the concepts of ventilatory and lactate threshold.. Sports Med.

[3] Hale T (2008). History of developments in sport and exercise physiology: A. V. Hill, maximal oxygen uptake, and oxygen debt.. J Sports Sci.

[4] Froböse I, Duesberg F, Verdonck A, Kurowski P (1990). Muscle stress of rehabilitation-oriented concentric and concentric-eccentric implemented isokinetic training.. Z Orthop Ihre Grenzgeb.

[5] Tomlin DL, Wenger HA (2001). The relationship between aerobic fitness and recovery from high intensity intermittent exercise.. Sports Med.

[6] Steinacker JM, Lormes W, Lehmann M, Altenburg D (1998). Training of rowers before world championships.. Med Sci Sports Exerc.

[7] Badtke G (1999). Lehrbuch der Sportmedizin.

[8] Kreckel V, Eysel P, König DP (2004). Injuries and muscle tightness in soccer.. Sportverletzungen und Sportschäden.

[9] Wienecke E (1998). Patient Bundesliga.

[10] Lötzerich H (1995). Hochleistungssport und Immunsystem.

[11] Liesen H, Baum M (1997). Sport und Immunsystem.

[12] Malm C, Lenkel R, Sjödin B (1999). Effects of eccentric exercise on the immune system in men.. European Journal of Applied Physiology.

[13] Ispirlidis I, Fatouros IG, Jamurtas AZ, Nikolaidis MG, Michailidis I (2008). Time-course of changes in inflammatory and performance responses following a soccer game.. Clin J Sport Med.

[14] Drexler H, Hornig B (1999). Endothelial dysfunction in human disease.. Journal of Molecular and Cellular Cardiology.

[15] Poveda JJ, Rientra A, Salas E, Cagigas ML, Lopez-Somoza C (1997). Contribution of nitric oxide to erxercise-induced changes in healthy volunteers; effects of acute exercise and long-term physical training.. European Journal of Clinical Investigation.

[16] Scott A, Khan KM, Roberts CR, Cook JL, Duronio V (2004). What do we mean by the term “inflammation”? A contemporary basic science update for sports medicine.. British Journal Sports Medicine.

[17] Thomas L (2005). Labor und Diagnose.

[18] Niess AM, Passek F, Lorenz I, Schneider EM, Dickhut HH (1999). Expression of antioxidant stress protein heme oxygenase-1 (HO-1) in human leukocytes.. Free Radic Biology Medicine.

[19] Wagener FADTG, Volk H.-D, Willis D, Abraham ND, Soares MP (2003). Different Faces of the Heme-Heme Oxygenase System in Inflammation.. Pharmacol Rev.

[20] Berndt G (2004). Antioxidative Wirkungen von NO-freisetzender NSAID in Endothelial- und Gastralzellen; Hämoxygenase1 als möglicher Mediator?.

[21] Doehner W, Schoene N, Rauchhaus M, Leyva-Leon F, Pavitt DV (2002). Effects of Xanthine Oxidase Inhibition with Allopurinol on Endothelial Function and Peripheral Blood Flow in Hyperuricemic Patients with Chronic Heart Failure.. Circulation.

[22] Wassermann K, Hansen JE, Sue DY, Stringer WW, Whipp BJ (2005). Principles of Exercise Testing and Interpretation; Including Pathophysiology and Clinical Applications..

[23] Kroidl RF, Schwarz S, Lehnigk B (2007). Kursbuch Spiroergometrie – Technik und Befundung verständlich gemacht.

[24] Doehner W, Rauchhaus M, Florea VG, Sharma R, Bolger AP (2001). Uric acid in cachectic and noncachectic patients with chronic heart failure – Relationship to leg vascular resistance.. Am Heart J.

[25] Duda GN, Taylor WR, Winkler T, Matziolis G, Heller MO (2008). Biomechnical, microvascular, and cellular factors promote muscle and bone regeneration.. Exerc Sport Sci Rev.

[26] Boutcher YN, Boutcher SH (2005). Limb vasodilatory capacity and venous capacitance of trained runners and untrained males.. Eur J Appl Physiol.

[27] Chen HI, Chang HR, Wu CY, Kao SJ, Wang D (2007). Nitric oxide in the cardiovascular and pulmonary circulation–a brief review of literatures and historical landmarks.. Chin J Physiol.

[28] Qi WN, Chen LE, Zhang L, Eu JP, Seaber AV (2004). Reperfusion injury in skeletal muscle is reduced in inducible nitric oxide synthase knockout mice.. J Appl Physiol.

[29] Jugdutt BI (2002). Nitric oxide and cardioprotection during ischemia-reperfusion.. Heart Fail Rev.

[30] Schellong SM, Ockert D, Zimmermann T (1998). Pathophysiology und Klinik der Ischämie – Reperfusionsschäden an der Skelettmuskululatur.. Vasa.

[31] Blaisdell FW (2002). The pathophysiology of skeletal muscle ischemia and reperfusion syndrome: a review.. Carciovasc Surg.

[32] Harre D (1982). Trainingslehre. 9.

[33] Hollmann W, Hettinger T (1990). Sportmedizin – Arbeits- und Trainingsgrundlagen. 3.

[34] Neumann G Sport und Medizin 2/1997.

